# Biomarkers Linked to Malnutrition Identified According to GLIM Criteria Among Older Community-Dwelling Adults: Results from the ilSIRENTE Study

**DOI:** 10.3390/nu17223543

**Published:** 2025-11-13

**Authors:** Hélio José Coelho-Júnior, Riccardo Calvani, Anna Picca, Matteo Tosato, Andrea Russo, Francesco Landi, Emanuele Marzetti

**Affiliations:** 1 Fondazione Policlinico Universitario Agostino Gemelli IRCCS, 00168 Rome, Italy; picca@lum.it (A.P.); matteo.tosato@policlinicogemelli.it (M.T.); andrea.russo1@policlinicogemelli.it (A.R.);; 2 Department of Geriatrics, Orthopedics and Rheumatology, Università Cattolica Del Sacro Cuore, 00168 Rome, Italy; 3 Department of Medicine and Surgery, LUM University, 70010 Casamassima, Italy

**Keywords:** undernutrition, blood markers, hemoglobin, anemia, kidney function, albumin, urema, C-terminal agrin fragment, potassium, calcium

## Abstract

**Objective**: This study aimed to examine the associations between malnutrition and circulating blood markers in older adults. **Methods**: We conducted a prospective cohort study on octogenarians residing in the mountain community of the Sirente geographic area in Central Italy. Data collection was conducted from December 2023 to September 2024. Malnutrition was defined based on the Global Leadership Initiative on Malnutrition (GLIM) criteria. A panel of blood markers was examined, and principal component analysis (PCA) was used to identify clusters of related molecules. Both unadjusted and adjusted binary logistic regression models were applied to investigate the associations between malnutrition and these molecular clusters. **Results**: Data from 196 older adults (mean age: 86.2 years) were analyzed. Malnutrition was positively associated with PC 2 (i.e., urea, c-terminal agrin fragment, and potassium) (odds ratio [OR] = 1.647, *p*-value: 0.039) and negatively associated with PC 3 (i.e., hemoglobin, hematocrit, and red blood cell count) (OR = 0.567, *p*-value: 0.022) and PC 4 (i.e., calcium, albumin, total protein levels, and HDL cholesterol) (OR = 0.607, *p*-value: 0.035). **Conclusions**: Findings of the present study suggest that different clusters of blood markers are associated with malnutrition in older adults. Specifically, malnutrition is associated with clusters related to kidney function, anemia, neuromuscular function, and nutrient availability. These associations likely reflect the underlying biological mechanisms contributing to the development of malnutrition in this population.

## 1. Introduction

Malnutrition (e.g., undernutrition) is a condition highly prevalent in older adults [1] characterized by insufficient intake or assimilation of nutrients, leading to significant changes in body composition and diminished biological function [2]. The presence of malnutrition in older adults has received considerable attention from international organizations, as its progression increases the risk of many negative outcomes, including falls, fractures, and death [3,4,5], and is associated with the development of cognitive impairment and depressive symptomatology [6,7].

The investigation of mechanisms and potential biomarkers underlying the development of malnutrition is a critical area of research. A recent pooled analysis of the literature identified several molecules significantly associated with malnutrition [8]. However, results were linked to considerable heterogeneity, with findings varying depending on the method used to define malnutrition [8].

The operationalization of malnutrition remains a significant challenge. The Global Leadership Initiative on Malnutrition (GLIM) [2] has proposed a paradigm that includes standardized criteria for diagnosing malnutrition, incorporating factors such as weight loss, insufficient food intake, and clinical signs, aiming to provide a more consistent approach to its identification and management. Nevertheless, to the best of our knowledge, no studies have yet investigated the mechanisms and biomarkers associated with this paradigm.

Hence, the present study examined data from the ilSIRENTE study to investigate the associations between blood-circulating molecules and malnutrition in older adults.

## 2. Materials and Methods

The present study drew upon data from the Aging and Longevity Study in the Sirente Geographic Area (ilSIRENTE) database [9]. This longitudinal cohort project was conducted within the mountainous Sirente region of Central Italy, located in L’Aquila (Abruzzo). The area encompasses 13 small rural municipalities situated between 800 and 1400 m above sea level and characterized by an agricultural-based economy. The ilSIRENTE initiative was jointly developed by the Department of Geriatrics at Università Cattolica del Sacro Cuore (Rome, Italy) and the teaching nursing facility Opera Santa Maria della Pace (Fontecchio, L’Aquila, Italy), with additional support from local authorities and primary care physicians of the Sirente Mountain Community.

The study complied with the principles of the Declaration of Helsinki and received approval from the Ethics Committee of Università Cattolica del Sacro Cuore (Rome, Italy). Written informed consent was obtained from all participants or, when required, from legally authorized representatives.

### 2.1. Participants

In October 2003, registry offices of the participating municipalities provided an official list of residents. Eligible individuals were those born before 1 January 1924 and living in the region at the time of baseline assessment. Of the 429 residents who met these criteria, 65 declined to participate, leaving 364 individuals for enrollment. For the current analysis, participants missing data on any biomarker (*n* = 122) or GLIM-based malnutrition criteria (*n* = 74) were excluded. Thus, 196 participants were included.

### 2.2. Data Collection

Baseline assessments began in December 2003 and ended in September 2004. All evaluations were performed during this period. Clinical interviews and functional testing were conducted at designated centers in each municipality, although home visits were arranged for individuals unable to travel due to health or logistical limitations. Information on medical conditions, medication use, and lifestyle habits was collected using validated questionnaires [9]. All assessments were carried out by a multidisciplinary team that included geriatric physicians, nurses, physiotherapists, medical residents, and medical students from the participating institutions, along with local primary care doctors. The ilSIRENTE database is overseen by the principal investigator (F.L.).

### 2.3. Malnutrition

Malnutrition was defined according to GLIM guidelines [2], requiring the presence of at least one phenotypic criterion and at least one etiologic criterion. Phenotypic indicators included: (a) unintentional weight loss: ≥5% in the last 30 days, or ≥10% in the last 180 days; (b) low body mass index (BMI): <22 kg/m^2^; and (c) low muscle mass: appendicular skeletal muscle (ASM) < 20 kg, for men, and <15 kg for women [10].

ASM was estimated using the calf-circumference-based equation developed by the COCONUT Study Group [11], which has been applied in previous research by our group [12,13] and others [14,15]. Etiologic criteria included: (d) decreased dietary intake, determined by answering “Yes, a little” or “Yes, a lot” to the question regarding reduced food consumption in the past year; (e) evidence of inflammation, defined as CRP ≥ 9 mg/LL [16].

### 2.4. Blood Markers

Fasting venous blood samples were collected from the median cubital vein into commercially available tubes and processed following standard laboratory procedures. Routine analyses were performed for hemoglobin, hematocrit, white blood cells, platelets, calcium, albumin, total protein, urea, potassium, lactate dehydrogenase, iron, creatine kinase, glucose, triglycerides, magnesium, sodium, amylase, phosphate, total cholesterol and its fractions (HDL, LDL), CRP, IL-6, and TNF-α using commercial kits (Olympus, Italy) on an Olympus 2700 analyzer (Center Valley, PA, USA). Serum C-terminal agrin fragment (CAF) was quantified using a commercial ELISA kit (NTCAF ELISA, Neurotune AG, Schlieren-Zurich, Switzerland) on a Spectramax 190 UV–VIS microplate reader (Molecular Devices, Sunnyvale, CA, USA). Free IGF-I and IGFBP-3 were assessed in triplicate using a certified radioimmunoassay (Diagnostic Systems Laboratories, Inc., Webster, TX, USA; distributed in Italy by Pantec S.r.l., Turin, Italy).

### 2.5. Covariates and Adjustment Variables

Height and weight were measured using a stadiometer and a medical scale, with participants lightly clothed and barefoot when feasible. BMI was calculated as weight (kg) divided by height squared (m^2^). Calf circumference was obtained from the dominant leg at the widest point between the knee and ankle while seated, recorded to the nearest 0.1 cm. Physical activity during the previous year was self-reported using predefined categories ranging from minimal activity (mostly bedridden) to high-intensity exercise or walking more than 5 km on at least five days per week. Definitions of low-, moderate-, and high-intensity activity were explained before questioning. Multimorbidity was defined as the presence of two or more chronic conditions, including obesity, cardiovascular diseases, stroke, heart failure, peripheral artery disease, hypertension, chronic lung diseases, osteoarthritis, diabetes, dementia, Parkinson’s disease, renal insufficiency, and cancer (excluding non-melanoma skin cancer), consistent with widely accepted standards in geriatric research [17]. Diagnoses were established using section J of the MDS-HC [18], incorporating self-reports, primary care records, physical examinations, and available laboratory and imaging data. Current smoking was defined as using tobacco at least weekly during the previous year. Educational attainment and time since the last hospitalization were assessed through items from sections BB and C of the MDS-HC [18]. Usual walking speed was measured over a 4 m walkway.

### 2.6. Statistical Analysis

Continuous variables were summarized as mean ± standard deviation (SD), and categorical variables as frequencies and percentages. Principal Component Analysis (PCA) was performed to identify patterns among the blood variables and to reduce dimensionality. Component retention was based on Kaiser’s criterion (eigenvalues > 1) and inspection of the scree plot. Six components were selected as they accounted for the major variability without overfitting. Components were extracted using principal axis factoring and Varimax rotation to enhance interpretability. Component loadings were examined to identify groups of biomarkers contributing to each factor. Associations between PCA-derived components and malnutrition were evaluated using binary logistic regression. Final models were adjusted for age, sex, BMI, physical activity in the past year, smoking status, educational level, time since last hospital admission, walking speed, and multimorbidity. Statistical significance was set at *p* < 0.05 (two-tailed). All analyses were conducted using SPSS version 23.0 (SPSS Inc., Chicago, IL, USA).

## 3. Results

### 3.1. Main Characteristics of Study Participants

Table 1 shows the main characteristics of study participants. Undernourished individuals were slower, older, had higher CAF and IL-6 levels, and lower total blood cholesterol, total blood proteins, albumin, calcium, iron, hemoglobin, hematocrit, and LDL levels, when compared to non-undernourished peers. The most prevalent conditions among those malnourished were hypertension (40.0%), osteoarthritis (24.4%), diabetes (20.0%), coronary arterial disease (17.8%), osteoporosis (15.6%), stroke (13.3%), heart failure (8.9%), and chronic obstructive pulmonary disease (11.1%). All other conditions were present in less than 10% of the participants (Table S1).

### 3.2. Identification of the Main Principal Components

**Figure 1:**
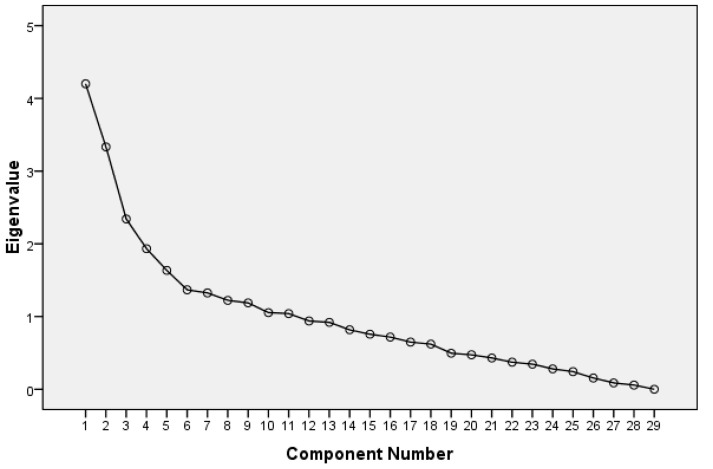
Scree plot exhibiting the eigenvalues for each individual PC.

Figure 1 and Table 2 show the eigenvalues of the PCs in descending order. Table 1 also includes the percentage of variance and the cumulative percentage of variance explained by each PC. Based on visual inspection, PCAs 1 to 6 were found to be the most representative, explaining a cumulative percentage of variance of 51.0%.

### 3.3. Associations Between PCAs and Malnutrition

Table 3 presents the results of the binary regression analysis between the representative PCs and malnutrition. The unadjusted analysis revealed that PCs 1, 2, 4, and 5 were significantly associated with malnutrition. After adjusting for covariates, only the associations with PCs 2 and 4 remained significant. Furthermore, PC3 became significantly associated. No other significant associations were observed.

### 3.4. Molecule Load

Table 4 presents the component matrix, which shows the correlation of each molecule with the respective PCAs. The molecules most associated with PCA 2 were urea (0.820), CAF (0.675), and potassium (0.494). For PCA 3, the molecules with the strongest associations were hemoglobin (−0.515), hematocrit (−0.514), iron (−0.350), and RBC (−0.416), while, for PCA 4, the most strongly correlated molecules were albumin (−0.436), total protein levels (−0.436), calcium (−0.223), and HDL (−0.223).

## 4. Discussion

The main findings of the present study suggest that different clusters of molecules are associated with malnutrition in older adults. Specifically, PC 2 (i.e., urea, CAF, and potassium) was significantly associated with malnutrition based on GLIM criteria. In contrast, negative associations were observed for PC 3 (i.e., hemoglobin, hematocrit, and RBC) and PC 4 (i.e., calcium, albumin, total protein levels, and HDL). These results likely reflect underlying biological mechanisms associated with the development of malnutrition.

The positive and significant associations between PC 2 and malnutrition might suggest a potential link between impairments in kidney function and the development of this condition among older adults. Other studies have found abnormalities in electrolyte balance in undernourished people [19]. A potential theoretical explanation for this scenario is that malnutrition might contribute to impairments in glomerular filtration rate (GFR) [20], which has a key role in the regulation of urea and potassium extracellular levels [21,22,23,24].

Urea is produced in the liver during the urea cycle as a mechanism to eliminate cytotoxic ammonia, in response to the demands of protein and nitrogen metabolism [24]. Increases in blood urea levels commonly reflect intrarenal causes, such as glomerulonephritis, chronic pyelonephritis, and toxic nephritis [24], and are partially associated with reduced GFR [23]. Impairments in GFR, lower clearance, are also one of the main causes of hyperkalemia [21,22,25], given that 90% of the excreted potassium exists through the kidneys [21].

The observation that CAF is elevated in PC 2 could be related to the role of agrin in the formation of the glomerular basement membrane [26], a specialized extracellular matrix structure within the glomerulus that is crucial for glomerular filtration. As such, increased CAF levels may reflect glomerular basement membrane degradation and indicate impairments in GFR [26]. Steubl et al. [27] found significant associations between GFR and systemic CAF levels in individuals with chronic kidney disease (CKD) [27]. Drey et al. [28] expanded on these findings, showing that CAF levels were significantly associated with the need for renal replacement therapy and the incidence of acute kidney injury. Furthermore, Lorenz et al. [29] reported that CKD patients with higher CAF levels had an increased risk of GFR decline.

Alternatively, some authors have proposed that the progressive reduction in kidney function might be linked with significant losses in muscle weight and mass, as those observed in malnutrition [28,30,31]. This assumption is important because CAF has been acknowledged as a marker of neuromuscular junction stability, with high levels being associated with low muscle mass and impaired physical function [26]. As such, the present findings may tentatively point to a possible interaction between malnutrition and a kidney–muscle axis among older adults. However, this interpretation remains highly speculative and should be confirmed in future longitudinal studies.

Our data might suggest that malnutrition is significantly associated with markers of anemia (i.e., PC 3) and with molecules directly linked to nutrient availability (i.e., PC 4). Numerous studies have found an increased prevalence of both low hemoglobin and low albumin levels in undernourished older adults from different settings (e.g., community-dwellers, hospitalized, and nursing-home residents) [32,33]. Specifically, studies have observed that nutrient deficiency and anemia occur simultaneously in one in every three older individuals [33], while hypoalbuminemia seems to be highly prevalent (>80%) in undernourished older adults [34].

A potential cause of this scenario is the insufficient intake of macronutrients frequently present in the diet patterns of people with malnutrition [33,35,36]. For instance, low protein intake might affect the production of hemoglobin, given that this protein contains four chains of amino acids, bonded to an iron ion, which, in turn, is connected to histidine, an essential amino acid [35]. Moreover, inadequate intake of both proteins and calories may affect hematopoiesis, independently of iron deficiencies and/or other hematopoietic factors [33,35].

Albumin is a hepatic protein produced by hepatocytes based on the availability of amino acids derived from dietary intake and muscle catabolism [37]. This molecule is often recognized as an indicator of nutritional status, due to its association with anthropometric markers and dietary changes [37]. Similar to hemoglobin, insufficient protein intake may reduce the availability of substrates required for albumin synthesis (i.e., amino acids) [37].

Notably, the inclusion of inflammation as a diagnostic criterion for malnutrition according to the GLIM criteria may have influenced our results, as inflammatory mediators are major regulators of both hemoglobin and albumin levels. Inflammation can reduce hemoglobin by impairing hematopoiesis [36] and decrease albumin by promoting capillary leakage, which moves hepatic proteins into the extravascular compartment [32].

The associations between malnutrition and other components of PC 4—calcium and HDL—might reflect, from a speculative point of view, a general state of insufficient nutrient intake. In fact, blood calcium levels are influenced by various nutritional factors that may be disordered in undernourished individuals, including the composition of plant nutrients and antinutrients, vitamin D intake, dietary protein consumption, and the intake of phytates (e.g., seeds, nuts, legumes) and oxalates (e.g., spinach, soy milk, potatoes) [38,39]. In addition, apparently healthy older adults often fail to reach the currently recommended calcium intake of 1000 mg daily, which suggests that a worse scenario probably exists in old people living with malnutrition.

Low HDL has been commonly observed in children with marasmus and kwashiorkor [40], though reports on older adults are still lacking. In this population, inflammation, combined with significant reductions in protein intake or a high adherence to specific dietary patterns (e.g., macrobiotic diets), has been suggested as a possible cause of this condition [40]. It is plausible that these conditions might also explain the results of the present study. More studies using more rigorous scientific methods are required to confirm this theoretical model.

The present study is not free of limitations. First, malnutrition was assessed using a modified version of the GLIM criteria. Secondly, ASM was estimated using calf circumference, rather than more accurate assessment tools. This approach was utilized because the use of more reliable techniques involves high expenses and requires the assistance of specific personnel and appropriate spaces, which hampers its implementation in epidemiological studies, such as the ilSIRENTE. However, although calf circumference is endorsed as an alternative method by the GLIM and ASM estimation was conducted using a validated formula, numerous studies have found that CC and gold-standard imaging techniques (e.g., DEXA) may not serve as equivalent measures of muscle mass [14,41,42,43]. This scenario raises the possibility that some individuals might have been misclassified. As such, the possibility that distinct results would be obtained through the estimation of body composition utilizing more reliable measurement methods cannot be ruled out. Third, covariates, including physical activity levels, were assessed using self-reported measures. Fourth, our small sample size prevented us from conducting sex-specific analysis. Fifth, important dietary aspects that might influence the associations examined in the present study, such as caloric and protein intake, were not recorded. For instance, inadequate intakes of iron, folate and B12 vitamins are common causes of anemia [44]. Furthermore, a more detailed examination of participants’ nutritional intake could contribute to understanding the associations between malnutrition and ingestion and absorption problems. Sixth, we evaluated a limited number of molecules, which do not encompass all possible and important mechanisms. Specifically, only IL-6 and TNF-α were examined to represent inflammation, while a plethora of possible mediators might be directly or indirectly linked to this condition [45,46,47]. This approach would also further clarify if associations observed in the present study are limited to some molecules and therefore to certain mechanisms or if they embrace distinct pathways. Seventh, future studies are required to examine if defining inflammation according to other GLIM proposed conditions (e.g., acute disease injury, chronic diseases) provides similar findings. Finally, we studied a cohort of relatively healthy, older adults living in a mountain region, and caution should be taken when generalizing these findings to other populations.

## 5. Conclusions

The findings of this study tentatively suggest that malnutrition in older adults may arise from the interplay of several interconnected biological systems rather than from a single isolated pathway. The positive association between malnutrition and a molecular cluster linked to kidney function (urea, CAF, and potassium) raises the possibility that early impairments in renal regulation or glomerular integrity might contribute to or result from nutritional decline in late life. Conversely, the negative associations observed with clusters reflecting anemia and nutrient availability (hemoglobin, albumin, calcium, and HDL) could indicate a general systemic insufficiency in nutrient intake, absorption, or utilization, potentially amplified by chronic low-grade inflammation.

Altogether, these findings may point toward a broader physiological network where deterioration in one system reinforces dysfunctions in others, ultimately culminating in malnutrition and frailty. While speculative, this hypothesis underscores the complexity of the biological underpinnings of malnutrition in advanced age. Future longitudinal and mechanistic studies integrating nutritional, metabolic, and inflammatory markers are warranted to clarify these interrelations and to determine whether interventions targeting renal health, muscle preservation, and nutrient metabolism might help prevent or mitigate malnutrition among older individuals.

## Figures and Tables

**Table 1:** Main characteristics of study participants (*n* = 196).

Variable	Non-Malnourished (*n* = 151)	Malnourished (*n* = 45)	*p*-Value
**Age (years)**	84.9 ± 4.5	**87.5 ± 4.9**	0.001
**Body Mass Index (kg/m^2^)**	26.0 ± 4.5	**23.8 ± 4.7**	0.006
**Men (%)**	37.7	**11.1**	0.001
**4 m walking test (m/s)**	0.54 ± 0.29	**0.25 ± 0.23**	0.001
**Comorbidities (%)**	73.2	**26.8**	0.340
**CAF (ng/mL)**	76.65 ± 39.40	**91.27 ± 44.46**	0.035
Glucose (mg/dL)	116.6 ± 40.9	120.8 ± 43.4	0.550
Urea (mg/dL)	46.7 ± 18.6	50.6 ± 24.3	0.256
**Cholesterol (mg/dL)**	197.6 ± 43.6	**180.2 ± 40.4**	0.018
Triglycerides (mg/dL)	149.1 ± 65.5	138.5 ± 65.4	0.344
HDL (mg/dL)	47.3 ± 14.2	43.7 ± 17.5	0.166
**LDL (mg/dL)**	132.7 ± 38.8	**116.8 ± 33.6**	0.014
Amylase (U/L)	74.9 ± 35.7	69.5 ± 39.9	0.388
Creatine kinase (U/L)	83.2 ± 49.9	69.7 ± 60.6	0.131
Lactate dehydrogenase (U/L)	318.5 ± 103.6	318.4 ± 98.9	0.999
**Total Proteins (g/dL)**	7.05 ± 0.52	**6.83 ± 0.56**	0.016
**Albumin (g/dL)**	4.23 ± 0.31	**4.10 ± 0.33**	0.016
**Calcium (mg/dL)**	8.99 ± 0.51	**8.68 ± 0.65**	0.001
Phosphorus (mg/dL)	3.32 ± 0.57	3.44 ± 0.80	0.252
**Iron (µg/dL)**	74.9 ± 34.0	**50.2 ± 30.4**	0.001
Sodium (mEq/L)	138.8 ± 4.7	139.2 ± 9.2	0.695
Potassium (mEq/L)	4.38 ± 0.48	4.32 ± 0.47	0.445
Magnesium (mEq/L)	1.91 ± 0.18	1.92 ± 0.22	0.870
**Hemoglobin (g/dL)**	13.64 ± 1.43	**12.38 ± 1.49**	0.001
**Hematocrit (%)**	40.66 ± 4.46	**37.94 ± 5.37**	0.001
Platelets (×10^3^/µL)	241,088 ± 131,361	256,294 ± 90,205	0.468
White blood cells (×10^3^/µL)	6923 ± 5265	6631 ± 1973	0.716
IGF-1 (ng/mL)	0.85 ± 0.73	0.88 ± 0.74	0.781
IGFBP-3 (ng/mL)	4506.8 ± 1417.6	4060.3 ± 1238.9	0.058
**IL-6 (pg/mL)**	2.38 ± 2.20	**4.42 ± 2.95**	0.001
TNF (pg/mL)	1.90 ± 2.58	2.56 ± 2.64	0.137

Values are mean ± SD or %. Significant differences (*p* < 0.05) are shown in bold. CAF = C-terminal agrin fragment; HDL = high-density lipoprotein; IGF = insulin-like growth factor; IL = interleukin; LDL = low-density lipoprotein; TNF = tumor necrosis factor.

**Table 2:** Total variance explained.

Component	Initial Eigenvalues		
	Total	% of Variance	Cumulative %
1	4.202	14.488	14.488
2	3.333	11.493	25.981
3	2.342	8.076	34.057
4	1.934	6.668	40.725
5	1.635	5.637	46.362
6	1.367	4.715	51.077
7	1.325	4.568	55.646
8	1.223	4.217	59.862
9	1.188	4.095	63.957
10	1.054	3.634	67.591
11	1.040	3.588	71.179
12	0.939	3.238	74.417
13	0.920	3.173	77.591
14	0.817	2.819	80.409
15	0.757	2.609	83.018
16	0.717	2.473	85.492
17	0.648	2.236	87.727
18	0.621	2.140	89.867
19	0.495	1.708	91.575
20	0.474	1.633	93.209
21	0.430	1.483	94.692
22	0.373	1.285	95.976
23	0.345	1.190	97.167
24	0.280	0.965	98.132
25	0.242	0.835	98.967
26	0.155	0.534	99.501
27	0.087	0.301	99.802
28	0.058	0.198	100.000
29	0.000	0.000	100.000

**Table 3:** Unadjusted and adjusted binary regression for the associations between principal components and malnutrition.

	OR	95% C.I. for EXP(B)		*p*-Value	Exp(B)	95% C.I. for EXP(B)		*p*-Value
		Lower	Upper			Lower	Upper	
PC1	0.565	0.389	0.821	0.003	0.764	0.491	1.190	0.234
PC2	1.649	1.178	2.308	0.004	1.647	1.024	2.649	0.039
PC3	0.747	0.521	1.071	0.113	0.567	0.349	0.921	0.022
PC4	0.483	0.335	0.697	0.000	0.607	0.381	0.966	0.035
PC5	0.614	0.426	0.884	0.009	0.812	0.550	1.201	0.298
PC6	1.041	0.749	1.447	0.810	0.970	0.630	1.492	0.889

**Table 4:** Molecule load.

	PC2	PC3	PC4
Calcium	0.291	−0.157	−0.223
Cholesterol	−0.252	0.587	0.005
Albumin	0.369	−0.262	−0.436
Proteins	0.369	−0.262	−0.436
Interleukin-6	0.263	0.059	−0.018
Low-Density Lipoprotein	−0.105	0.408	−0.087
Insulin-Like Growth Factor Binding Protein 3	0.109	0.223	0.309
Osmolality	0.825	−0.147	0.022
Urea	0.820	0.023	0.301
C-terminal agrin fragment	0.675	0.052	0.362
Potassium	0.494	−0.044	0.074
Hemoglobin	−0.374	−0.515	0.336
Hematocrit	−0.314	−0.514	0.349
Red Blood Cells Count	−0.144	−0.416	0.591
Lactate Dehydrogenase	0.133	0.444	0.154
Iron	−0.350	−0.134	−0.042
Creatine Kinase	0.105	0.319	0.034
Glucose	0.101	−0.313	0.181
Triglycerides	0.020	0.167	0.280
Platelets	0.042	0.036	0.054
High-Density Lipoprotein	−0.206	0.204	−0.289
Magnesium	−0.057	0.323	0.243
Sodium	0.319	−0.178	−0.233
Insulin-Like Growth Factor 1	0.006	0.319	0.273
Amylase	0.201	−0.019	0.094
Mean Corpuscular Volume	−0.103	0.028	−0.356
White Blood Cells Count	−0.012	0.041	0.129
Phosphorus	0.315	0.359	0.145
Tumor Necrosis Factor	0.216	−0.181	−0.059

## Data Availability

The data presented in this study are available on request from Francesco Landi due to ethical reasons.

## Supplementary Materials

The following supporting information can be downloaded at: https://www.mdpi.com/article/10.3390/nu17223543/s1, Table S1. Prevalence of major comorbidities by nutritional status (GLIM).
