# Supplementary material for: Biomarkers Linked to Malnutrition Identified According to GLIM Criteria Among Older Community-Dwelling Adults: Results from the ilSIRENTE Study
Source: Nutrients. 2025 Nov 13;17(22):3543. doi: 10.3390/nu17223543 (PMC12655322; doi:10.3390/nu17223543)
Supplement: Supplementary file 1 [file nutrients-17-03543-s001.zip › nutrients-3951434-supplementary.pdf]

Table S1. Prevalence of major comorbidities by nutritional status (GLIM).

Condition	Non-malnourished (n = 151)	Malnourished (n = 45)
**Hypertension**	82 (54.3%)	**18 (40.0%)**
**Osteoarthritis**	34 (22.5%)	**11 (24.4%)**
**Diabetes mellitus**	8 (5.3%)	**9 (20.0%)**
**Coronary artery disease**	16 (10.6%)	**8 (17.8%)**
**Osteoporosis**	11 (7.3%)	**7 (15.6%)**
**Stroke**	2 (1.3%)	**6 (13.3%)**
**COPD**	17 (11.3%)	**5 (11.1%)**
**Heart failure**	5 (3.3%)	**4 (8.9%)**
**Renal insufficiency**	2 (1.3%)	**1 (2.2%)**
**Peripheral vascular disease**	5 (3.3%)	**0 (0.0%)**
**Cancer**	9 (6.0%)	**0 (0.0%)**
**HIV infection**	0 (0.0%)	**0 (0.0%)**
**Pneumonia**	0 (0.0%)	**0 (0.0%)**
